# In memory of Prof. Dr. med. Rolf Sauer: September 19, 1939–April 20, 2026

**DOI:** 10.1007/s00066-026-02555-8

**Published:** 2026-06-01

**Authors:** Rainer Fietkau, Jürgen Dunst, Dirk Vordermark, Wilfried Budach, Claus Rödel

**Affiliations:** 1 University Hospital Erlangen, 91054 Erlangen, Germany; 2 Department of Radiation Oncology, Campus Kiel, University Hospital Schleswig-Holstein, 24105 Kiel, Germany; 3 Department of Radiotherapy and Radiation Oncology, Martin Luther University Halle-Wittenberg, 06120 Halle, Germany; 4 German Society for Radiation Oncology, 10117 Berlin, Germany; 5 Department of Radiotherapy and Oncology, University Hospital Frankfurt, 60590 Frankfurt, Germany

It is with profound sorrow, yet deep gratitude, that we bid farewell to Prof. Rolf Sauer, MD, the founding director of the Department of Radiation Oncology at Erlangen University Hospital, who passed away at the age of 86. With his death, we lose one of the most influential figures in German and international radiation oncology—a physician, scientist, teacher, and an individual of extraordinary distinction.

**Figure 1:**
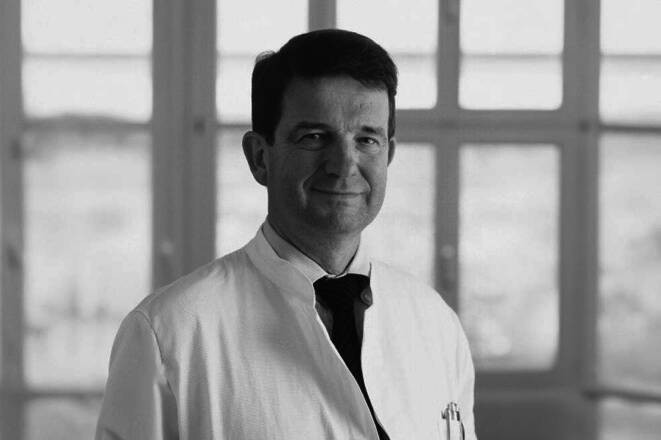
Prof. Dr. med. Rolf Sauer. (Foto: Uniklinikum Erlangen/Knuth Pflaumer)

## Professional career—From Hamburg and Basel to Erlangen

Rolf Sauer was born on September 19, 1939, in Hamburg. He studied medicine in Hamburg and Vienna and earned his doctorate in Hamburg. His academic journey led him to Basel, where he began as a research assistant at the Institute of Physiology and completed his specialist training in radiology and radiation oncology. In 1976, he received his habilitation at the University of Basel with a thesis on dose-rate effects on pluripotent hematopoietic stem cells in mice—a work that already reflected his lifelong commitment to bridging the gap between basic research and clinical application.

In 1977, he was appointed to the first chair of radiation therapy in Bavaria at Friedrich-Alexander University Erlangen-Nuremberg—the fourth such chair in Germany. From modest beginnings, he shaped over three decades a department that would grow into one of Europe’s leading centers in radiation oncology, a clinic of international renown that continues to bear his intellectual and human spirit.

## Medical director and architect of the University Hospital Erlangen

From 1996 to 2006, Prof. Sauer served as Medical Director and Chairman of the Board of University Hospital Erlangen. In this role, he guided the institution through a decisive period of transformation, strengthening its strategic foundations and advancing a vision of modern, interdisciplinary patient care. The structures he initiated and the buildings realized under his leadership remain an enduring testimony to his foresight.

He was a tireless advocate for the development of integrated oncology. Under his guidance, patient care evolved from parallel, isolated disciplines to coordinated, interdisciplinary collaboration. Surgery, medical oncology, radiation oncology, pathology, and allied disciplines were brought together in structured treatment pathways—always with the patient at the center.

Of particular importance to him was the establishment of specialized cancer centers. He recognized early that the concentration of expertise, guided by clear quality standards and continuous evaluation, would be essential to improving outcomes in cancer care. For him, this was never merely organizational—it was a deeply medical and ethical mission: better outcomes for patients through excellence.

The impact of this vision extended far beyond Erlangen. His pioneering role in advancing center-based oncology helped shape the development of cancer care structures throughout Germany and contributed significantly to establishing certification as a hallmark of quality in oncology.

## A pioneer of interdisciplinary oncology

What distinguished Rolf Sauer was his unwavering conviction that oncology can only succeed through collaboration. Long before it became standard practice, he developed multimodal treatment concepts in which surgical, medical, and radiation oncology were not isolated, but worked effectively together—an approach that also strengthened radiation oncology as an equal and indispensable pillar of comprehensive cancer care.

With the founding of the Erlangen Tumor Center, which he chaired until 1992, he created an institutional embodiment of this vision. The Erlangen Department of Radiation Oncology became not only a place of clinical excellence, but also a role model for modern, interdisciplinary oncology.

## Landmark contributions to oncology

Rolf Sauer’s scientific legacy finds its most enduring expression in the landmark multimodal trial CAO/ARO/AIO-94 on the treatment of locally advanced rectal cancer. At a time when postoperative chemoradiotherapy was the accepted standard, he and his colleagues questioned this established practice to explore a new paradigm: neoadjuvant treatment prior to surgery. The results, published in 2004, set a new standard of care worldwide—demonstrating improved local control, better tolerability, and higher rates of sphincter preservation. For patients, this translated into fewer recurrences, fewer permanent stomas, and a markedly improved quality of life.

Yet the significance of this work extends far beyond rectal cancer. It established neoadjuvant therapy as a guiding principle of modern oncology—a shift in thinking that continues to shape treatment strategies across numerous tumor entities worldwide. In addition, his scientific legacy stretched across such key innovations as breast-conserving approaches in breast cancer, organ-preserving strategies in bladder cancer and the combination of radiotherapy with hyperthermia—always guided by the dual aim of cure and improved quality of life. Rolf Sauer was not only a contributor, but a pioneer of new therapeutic paradigms.

## Service to the scientific community

His commitment to interdisciplinarity was equally reflected in his extensive service to scientific societies and committees. He served as Chair of the Radiation Oncology Section of the German Radiological Society (1979–1985) and founded the Working Group for Clinical Cancer Research of the ARO (Association of Radiation Oncology) of the German Cancer Society in 1986, leading it until 1993. From 1979 to 1998, he was a member of the Protocol Review Committee of the Federal Ministry of Research and Technology and the German Cancer Society. In 2007, he founded the “Atzlesberg Circle” for the implementation of clinical studies on hyperthermia, which he led until 2015.

Education and the exchange of knowledge were central to his mission. The “Erlangen Weiterbildung,” which he initiated in 1981, and the “Rothenburger Tage” became enduring forums for interdisciplinary dialogue and continuing education. He was repeatedly called upon to organize major national congresses (including the Bavarian Radiology Congress in Nuremberg 1996, the 4th Congress of the German Society for Radiation Oncology in Nuremberg 1998, and the 20th Annual Meeting of the German Society for Senology in Nuremberg 2002), reflecting the high esteem in which he was held across disciplines.

## Editor-in-chief of “Strahlentherapie und Onkologie”

As editor-in-chief of “Strahlentherapie und Onkologie” (1993–2015), he developed the journal into an internationally recognized scientific publication. The journal became the official publication of DEGRO in 1994 and has since been adopted by seven European radiation oncology societies, including ÖGRO and SASRO. Founded in 1912 as the oldest scientific journal in oncology, “Strahlentherapie und Onkologie” was thus successfully guided into the future under his leadership.

## Honors and recognition

The many honors bestowed upon him reflect the breadth and depth of his contributions; among them membership in the “Sächsischen Akademie der Wissenschaften” (1992), “Deutschen Akademie der Naturforscher Leopoldina” (2001), “Bundesverdienstkreuz 1. Klasse” (2002), “Deutscher Krebspreis” (2004), “Johann-Georg-Zimmermann-Medaille” (2005/2006), “Röntgen-Plakette” (2007), and “Karl-Heinrich-Bauer-Medaille” (2013).

In recognition of his scientific achievements, he was granted honorary memberships in numerous professional societies, such as the Hungarian Society of Radiation Oncology (1999), the Radiological Society of North America (2001), the Hungarian Cancer Society (2001); the Bavarian Radiological Society (2004), and the DEGRO (2009).

## Teacher, mentor, and friend

Beyond all titles and achievements, Rolf Sauer trained a generation of radiation oncologists and created what is often referred to as “Erlanger Schule”. For him, education meant not only imparting knowledge but also shaping a professional ethos that places humanity at the center of patient care—especially in a highly technological field such as radiation oncology. He fostered the conviction that a radiation oncologist not only treats but actively shapes the course of care—not waiting for others to decide, but advancing treatment within an interdisciplinary framework.

We saw him as a discussion partner whose experience and judgment were always highly valued. He could listen, not just lecture. He could persuade, not just command. Rolf Sauer was a man of principle—both literally and figuratively: Upright, clear, direct, never hurtful, never lacking in respect. He demanded a lot—most of all from himself.

Rolf Sauer shaped radiation oncology in Germany in a lasting and profound way—through institutions, through ideas, and through people. His legacy endures and lives on. We bow our heads in respect for a life devoted to medicine, science, and humanity. We will honor his memory—in the clinic he founded, in the field he shaped, and in the people who were fortunate enough to know him.

Our deepest sympathy goes to his wife, his three children, and all who mourn his loss.

Prof. Dr. Rainer Fietkau, Prof. Dr. Jürgen Dunst, Prof. Dr. Dirk Vordermark, Prof. Dr. Wilfried Budach, Prof. Dr. Claus Rödel

Erlangen, April 2026

## Data Availability

All data supporting the findings of this work are included in the article.

